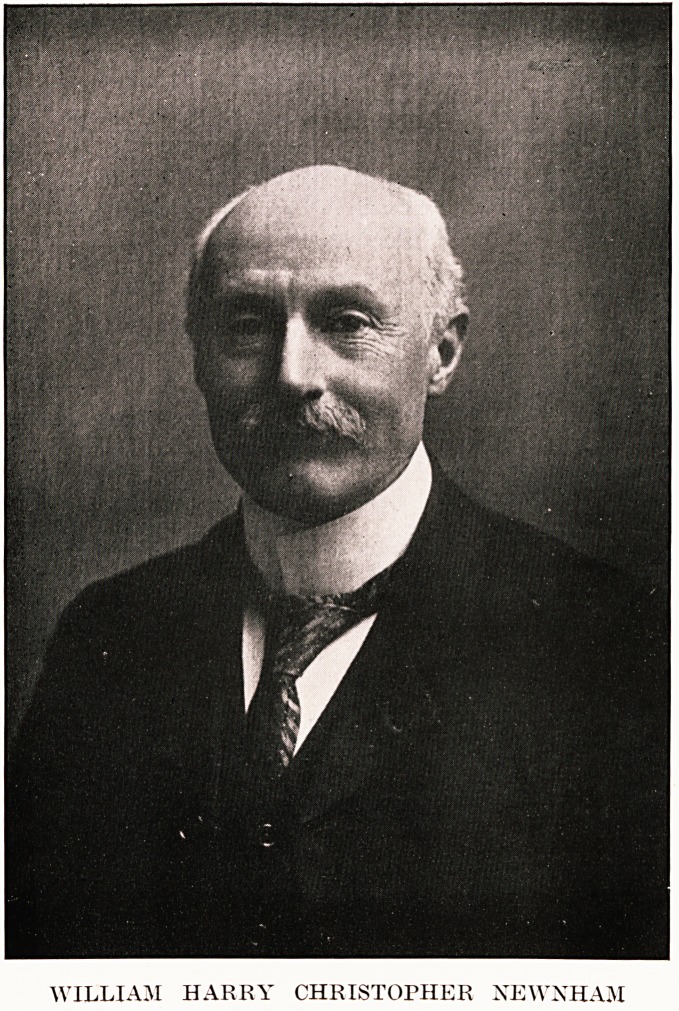# W. H. C. Newnham

**Published:** 1941

**Authors:** 


WILLIAM HARRY CHRISTOPHER NEWNHAM

August, 1922, the Medical School became entirely incorporated with the University.

Thus the inter-regnum when Francis acted as Vice-Chancellor in 1921–22, is perhaps the most memorable chapter in the history of that office in the eyes of the Medical Faculty of the University.

His death is deeply regretted and our sympathy is offered to his widow.

### Obituary

### W. H. C. NEWNHAM, M.A., M.B., M.R.C.S.

WE regret to announce the death of Dr. William Harry Christopher Newnham, which took place at Clifton on 11th January; he was in his eighty-fifth year. For many years Dr. Newnham was one of Bristol's leading medical men. He was born at Wolverhampton, where his father practised for many years. From Wolverhampton Grammar School he went up to Gonville and Caius College, Cambridge, in 1874, graduating B.A. in 1878 and proceeding to M.A., M.B., in 1883. He had obtained the M.R.C.S. diploma in 1880. After leaving Cambridge he became a student at Guy's Hospital, where he held the posts of house-surgeon and resident obstetric officer. His next appointment was resident medical officer at the Evelina Hospital for Children. He went to Bristol as house-surgeon at the Bristol General Hospital in 1887. At the end of three years, having decided to specialize in obstetrics and gynæcology, he was appointed to the post of assistant obstetric physician to the hospital, and on the death of Dr. Aust Lawrence in 1897 he was appointed obstetric physician, retiring under the age limit in 1923. He soon acquired an extensive practice in his speciality, and played a prominent part in the development of the obstetrical and gynæcological department of the Bristol General Hospital.

Dr. Newnham was devoted to his hospital work; he took the greatest interest in his patients, usually visiting them every day. Even after his retirement he frequently came down and did a round of the wards, and was always warmly welcomed by the patients and hospital staff. Dr. Newnham was a former president of the Bristol Medico-Chirurgical Society, a Fellow of the Royal Society of Medicine, and a Member of the British Medical Association for forty-nine years. From 1917 to 1920 he was Churchwarden of Clifton Parish Church, for he took a deep interest in the parish and its Church throughout the whole time of his residence in Clifton. His principal relaxations were the theatre and a game of bridge. He married a daughter of the late Mr. William C. Beloe of Clifton, and has two daughters. He suffered a grievous loss when Mrs. Newnham died several years ago, from which he never really recovered. In his latter years he was overtaken by blindness, but he nevertheless went about alone and confidently. He admitted his disability to his friends, begging them not to pass him in the street because he could not recognize them and might seem wanting in courtesy. Until a few weeks before his death he was very active and took the greatest interest in everything. He will be greatly missed by a large circle of friends in Clifton.

[Reprinted, by kind permission, from *The British Medical Journal*, February, 1941.]

## Figures and Tables

**Figure f1:**